# Chicago Medical Society

**Published:** 1886-06

**Authors:** 


CHICAGO MEDICAL SOCIETY.

An excellent precedent in the conduct of debates before the CHICAGO MEDICAL SOCIETY has been recently established by the PRESIDENT.

Special invitations, requesting participation in debate, have been sent to the various members of the local profession, who are supposed to be most conversant with the topic under consideration. Irrelevant discussion is thus cut off to a very great extent, and the author of a carefully prepared paper secures the critical attention of a jury of his peers.

The interesting character of the transactions at the last regular meeting,—when Professor A. Reeves Jackson's paper on "The Intra-Uterine Stem in the Treatment of Flexions" was read,—was due largely to this welcome innovation.

